# A review of neuroprotective properties of *Centella asiatica* (L.) Urb. and its therapeutic effects

**DOI:** 10.1080/07853890.2025.2559122

**Published:** 2025-09-11

**Authors:** Jiamei Jiang, Ranran Han, Honglei Ren, Yang Yao, Wei Jiang

**Affiliations:** a Department of Neurology, Tianjin Neurological Institute, Tianjin Medical University General Hospital, Tianjin, China; b School of Clinical Medicine, Tsinghua Medicine, Tsinghua University, Beijing, China

**Keywords:** *Centella asiatica*, oxidative stress, neuroinflammation, neurodegeneration, brain injury

## Abstract

**Introduction:**

*Centella asiatica* (L.) Urb., a traditional medicinal plant widely distributed in Southeast Asia, has been demonstrated to possess significant neuroprotective effect. However, the pharmacological and molecular mechanisms of *C. asiatica* (CA) in treating neurological diseases remain to be further explored. This review synthesizes evidence on CA’s pharmacokinetics, antioxidant/anti-inflammatory mechanisms, and therapeutic potential in various neurological disorders.

**Discussion:**

Evidence shows CA can mitigate oxidative stress, inflammation, mitochondrial dysfunction, and neuronal apoptosis—key mechanisms underlying these conditions. In epilepsy models, asiatic acid suppresses glutamate release, improves synaptic and mitochondrial function, and reduces neuronal damage. In neurodegenerative diseases, CA extracts enhance memory and cognitive functions by activating antioxidant response pathways and protecting hippocampal mitochondria from oxidative stress. CA extracts also display anticonvulsant effects without major toxicity. Against Bisphenol A-induced neurotoxicity, CA alleviates oxidative stress and inflammation while restoring mitochondrial function. For radiation-induced brain injury, CA improves cognition and memory *via* antioxidant pathways and anti-inflammatory effects. In cerebral ischemia-reperfusion injury, asiaticoside reduces oxidative stress and neuroinflammation through NOD2/MAPK/NF-κB pathway modulation and microglial regulation. Recent research suggests that CA also plays a significant role in alleviating symptoms of anxiety and depression. The plant’s active components have been found to regulate neurotransmitter activity, reduce oxidative stress, and modulate the hypothalamic-pituitary-adrenal (HPA) axis.

**Conclusions:**

CA demonstrates broad neuroprotective potential, offering multi-target benefits with relatively low toxicity. These findings support its development as a novel therapeutic agent for neurological disorders. Further clinical research is warranted to confirm safety, optimize dosing, and translate preclinical results into effective human treatments.

## 1. Introduction

*Centella asiatica* (L.) Urban is one of the traditional medicines widely reported to effectively attenuate neurological problems like neuroinflammation and neurodegeneration. *Centella asiatica* (CA) is a prostrate, perennial herbaceous creeper belonging to the family Apiaceae which thrives abundantly in moist areas at altitudes up to 1800 m in tropical and subtropical regions [1,2]. CA has long been used as a medicinal herb since ancient times by indigenous people in India, Sri Lanka, Madagascar, South Africa, the South Pacific, and Eastern Europe, primarily for treating high blood pressure, enhancing memory, and promoting longevity [3]. The extensive pharmacological properties of CA and its extracts was also investigated in a growing number of scientific studies. Previous articles reported that extracts of CA can heal wounds, burns, and ulcerous on neurological [4], cardiovascular [5], gastrointestinal [6], and respiratory [7] diseases, as well as exhibiting anti-breast cancer properties [8].

CA exhibits broad neuropharmacological effects, primarily attributed to its significant antioxidative, anti-inflammatory, and anti-apoptotic properties [9]. One notable example is asiaticoside-D, a derivative of asiaticoside, which is a major component isolated from CA extracts. This compound has been shown to counteract the rotenone-induced increase in reactive oxygen species (ROS) levels in the brain, a critical factor in combating neurodegenerative diseases like Parkinson’s disease [10]. CA’s antiepileptic effects have also been demonstrated in several studies, with one suggesting that CA can alleviate seizure-induced cognitive impairment by reversing neuronal damage through the restoration of mitochondrial function [11]. In addition, recent studies showed that CA possesses neuroprotective effects against transthyretin (TTR) amyloidosis by inhibiting pathological TTR fibril formation and reducing cytotoxicity from amyloid aggregates. These findings highlight CA’s potential as a therapeutic candidate for this currently untreatable neurodegenerative condition [12–14].

Furthermore, a study on the Blood-Brain Barrier (BBB) permeability of CA’s bioactive components—asiaticoside, madecassoside, and asiatic acid—revealed that these compounds have a high capability to cross the BBB. This finding further supports CA’s potential as an ideal candidate for developing new, more effective therapeutic strategies for neurological disorders [15]. The complex cellular and molecular mechanisms involved, along with CA’s other medicinal properties, will be further discussed in the subsequent sections of this article.

In this review, we summarize the biochemical properties and neuroprotective effects of CA and its active compounds. We systematically analyze the bioactivity and physicochemical properties of CA and its derivatives, providing insights into their absorption and metabolic mechanisms - aspects that were insufficiently addressed in previous studies. Furthermore, while the neuroprotective effects of CA in various neurological disorders have been widely reported, we integrate network pharmacology studies to elucidate the associated signaling pathways and molecular mechanisms. Finally, given CA’s notable blood-brain barrier (BBB) permeability, we discuss the underlying physicochemical mechanisms and potential drug delivery strategies.

## 2. Methodology

This review was conducted based on a comprehensive survey of previous studies, with a primary focus on CA and its neuroprotective effects. Information on molecular weight and chemical properties was obtained from PubChem. Relevant literature was selected from electronic English-language databases, such as PubMed, Google Scholar, Web of Science, Elsevier, and others, using the following keywords: ‘*C. asiatica*’, ‘oxidative stress’, ‘neurodegeneration’, ‘neuroinflammation’, ‘brain injury’, ‘asiatic acid’, ‘neuroprotection’, ‘blood-brain barrier’, and related terms. Only published studies written in English that specifically addressed the neuroprotective properties and potential therapeutic effects of CA and its bioactive components on various neurological diseases were included. Studies were excluded if they lacked accessible sources, contained unpublished data, provided no supporting evidence, or were irrelevant to the neuroprotective effects of CA and its extracts.

## 3. Absorption, distribution profiles, and bioavailability of secondary bioactive metabolites of CA

**Figure 1.**
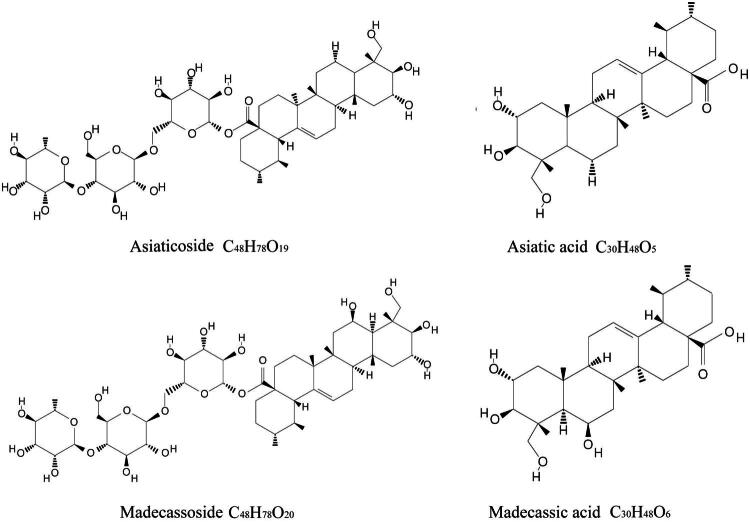
Chemical structures and molecular formulas of four triterpene saponins of CA.

Asiatic acid, madecassic acid, asiaticoside, and madecassoside are four triterpenoid components of CA which are the principle saponin-containing substances of major therapeutic interest [16,17]. Their chemical structures and molecular formulas are described in Figure 1.

Asiaticoside (AS) (Pubchem CID: 11954171) has a molecular weight of 959.1 g/mol and a molecular formula of C_48_H_78_O_19_. Asiaticoside effectively reduces oxidative stress and apoptosis in epithelial cells [18] and neurons as well as related anti-inflammatory mechanisms [19]. Madecassoside (MS) (Pubchem CID: 45356919) has a molecular formula of C_48_H_78_O_20_, and a molecular weight of 975.1 g/mol. Despite its extremely low bioavailability as a triterpenoid saponin, madecassoside demonstrates significant therapeutic effects in collagen-induced arthritis (CIA) rats after oral administration [20]. Asiatic acid (AA) (Pubchem CID:119034) has a chemical formula of C_30_H_48_O_5_ and a molecular weight of 488.7 g/mol. Asiatic acid is an aglycone form of asiaticoside, which exerts anticancer effects in breast cancer cells [21], and attenuates inflammatory response in brain injury mice [22]. Madecassic acid (MA) (Pubchem CID: 73412) has a molecular weight of 504.7 g/mol and a molecular formula of C_30_H_48_O_6_. Similar as asiatic acid, madecassic acid is the aglycone form of madecassoside.

The bioactive properties of CA are attributed to its triterpene saponins, which are common secondary plant metabolites synthesized *via* the isoprenoid pathway. This pathway produces a hydrophobic triterpenoid structure (aglycone) linked to a hydrophilic sugar chain (glycone) [23]. Several factors can influence the content of pentacyclic triterpenes in CA. A previous study demonstrated that the leaves of the plant contain the highest concentration of pentacyclic triterpenes, totaling 19.5 mg/g. Additionally, factors, such as the place of cultivation and the harvesting period can significantly alter the triterpene content in CA extracts [24].

The absorption and tissue distribution profiles of madecassoside and asiaticoside are quite similar, with both triterpenoids being extensively distributed in the brain, stomach, and skin within 1 h after dosing, despite their low oral bioavailability of <1% [25]. The levels of asiatic acid and madecassic acid in mice were found to increase significantly in the plasma, brain, heart, liver, kidney, colon, and bladder after 8 weeks of oral administration, compared to 4 weeks of administration [26]. Furthermore, ECa233, a standardized *C. asiatica* extract containing ≥80% triterpenoid glycosides (madecassoside:asiaticoside ratio∼1.5:1.2), demonstrates distinct pharmacokinetic properties. Intravenous administration in rats showed rapid tissue distribution, with the highest accumulation in the spleen, followed by extensive metabolism. Notably, madecassoside and asiaticoside underwent interconversion and hydrolytic cleavage to their aglycones (madecassic acid and asiatic acid) [27]. Research shows that CA’s bioactive components are rapidly absorbed and metabolized. Asiatic acid, for instance, reaches peak plasma concentration (*C*_max_) within 30 min and has a short elimination half-life (*t*_1/2_ = 0.348 h), indicating extensive first-pass metabolism [28]. An liquid chromatography-electrospray ionization-mass spectrometry (LC-ESI-MS) study revealed madecassoside’s wide tissue distribution (primarily liver/kidney), hepatobiliary excretion, and enterohepatic recirculation in rats. Its metabolism involves stepwise hydrolysis which indicates possible drug interactions [29].

Despite the extensive pharmacological effects of CA and its extracts, the low intestinal absorption and bioavailability of them limits their applications in the medical field [30,31]. Pentacyclic triterpenoids are the main bioactive components in CA, of which the poor transmembrane permeability may result in the low intestinal absorption after oral administration. To address this problem, Wang et al. developed liposomes loaded with Centella Total Glucosides (CTG) which are the total saponins in CA to promote the absorption in the ileum thereby improving their therapeutic potentials with higher bioavailability [32].

Notably, the poor water solubility of the pharmacologically active components of CA due to their chemical structures and properties also results in low bioavailability. The main four bioactive compounds of CA—including asiatic acid, asiaticoside, madecassic acid, and madecassoside—are pentacyclic triterpenoids possess relatively large molecular weights (e.g. asiaticoside ∼ 959.1 g/mol) which limit their ability to freely diffuse through aqueous media and reduce their systemic absorption [33]. Besides, the main structure of triterpenoids (the steroidal core) is highly hydrophobic, leading to their extremely poor water solubility [34].

Pharmacosomes appear to be among the promising approaches explored recently to improve drugs with limited water solubility and bioavailability. They are amphiphilic drug-phospholipid complexes that form highly ordered assemblies of concentric lipid bilayers in aqueous solutions, which can increase drug solubility in aqueous, as well as enhancing drug bioavailability significantly after oral administration [35]. A phospholipid-based complex of standardized *Centella* extract (SCE) was developed to obtain a significantly higher (12-fold) aqueous solubility and hence to improve the systemic absorption and bioavailability of CA’s constituents, which exhibited positive effects in improving the spatial learning and memory in aged mice [36]. A study by Boonyarattanasoonthorn et al. developed a newly standardized CA extract (Centell-S) with improved water solubility and investigated the pharmacokinetic profiles of its bioactive compounds. The enhanced water solubility of Centell-S led to higher oral bioavailability of triterpenoid saponins, such as madecassoside and asiaticoside, compared to ECa 233 in orally administered beagle dogs [37]. In conclusion, CA extracts with improved water solubility demonstrate increased oral bioavailability, offering us new perspectives for the future development of pharmaceutical products derived from CA. Future research should prioritize improving the low bioavailability of these medicinally valuable large particles due to their poor intestinal absorption and water solubility.

Nalinratana et al. explored the differential signaling modulation of madecassoside, asiaticoside, and their aglycones in Neuro-2a cells. The study revealed that the percentage of neurite-bearing cells (%NBC) and neurite length were significantly increased by madecassoside (MS) and asiaticoside (AS), exhibiting greater potency compared to madecassic acid (MA) and asiatic acid (AA). Additionally, MS and AS induced persistent phosphorylation of extracellular signal-regulated protein kinases 1 and 2 (ERK1/2), leading to CREB activation, whereas their aglycone forms (MA and AA) only triggered transient ERK1/2 signaling. Interestingly, the suppression of ERK phosphorylation did not result in similar CREB inhibition and neurite outgrowth, indicating that ERK activation was not associated with MA- and AA-induced neurite outgrowth [38]. In a subsequent study in 2019, also by Nalinratana et al., the neuritogenic and neuroregenerative mechanisms of AS and AA were further investigated, focusing on ERK1/2 and protein kinase B (Akt) signaling. Their findings suggest that, although both AS and AA exhibit neuritogenic properties in Neuro-2a cells, only AS modulates this effect through TrkA receptor signaling, whereas AA regulates different upstream molecules [39]. These intriguing results highlight that, while all four compounds demonstrate neuroprotective properties, their specific cellular and molecular mechanisms may differ significantly. Consequently, further investigation into the biochemical mechanisms underlying the actions of asiaticoside, madecassoside, asiatic acid, and madecassic acid remains necessary.

Here, we briefly summarize the fundamental biochemical properties of the four major secondary metabolites of CA, along with their absorption and distribution profiles, and discuss their differences in modulating neuritogenic signaling pathways. The subsequent section will focus on the extensive anti-oxidative, anti-apoptotic, and anti-inflammatory effects of CA, both *in vivo* and *in vitro*, and will explore the potential of CA and its derivatives as therapeutic agents for the treatment of various neurological diseases.

## 3. Neuroprotective effects: anti-oxidative, anti-inflammatory, and anti-apoptosis properties of CA

**Figure 2.**
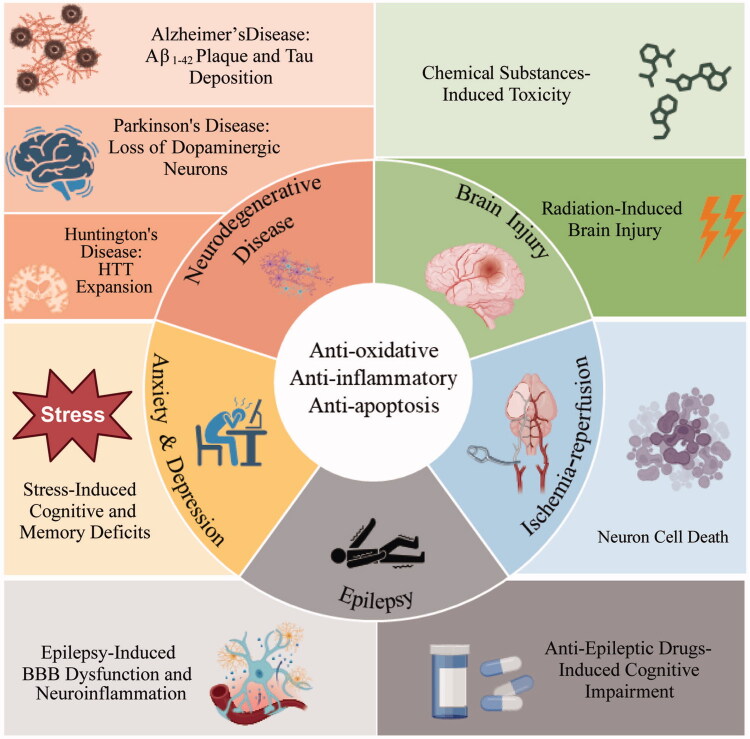
The anti-oxidative, anti-inflammatory, and anti-apoptosis neuroprotective effects of CA and its therapeutic effects. Created with BioRender.com.

The protective effects of CA reducing oxidative stress and neuroinflammation and inhibiting cell apoptosis are widely discussed in previous studies and summarized in Figure 2. A comparative analysis between *Bacopa monnieri* and CA revealed their similar mechanisms as memory enhancers and neuroprotectants, the former of which has long been recognized for its role in treating neurological disorders characterized by oxidative stress. These common findings suggest that the neuroprotective properties of both herbal medicines may be attributed to shared potential protein targets [40,41].

In a study using a 6-hydroxydopamine (6-OHDA)-induced PC12 cell injury model, Wu et al. screened the bioactive constituents isolated from the 80% methanol extract of CA. Their results demonstrated that several of the isolated compounds exhibited significant neuroprotective effects. Notably, the most effective compound, compound 3, attenuated cell apoptosis, enhanced the mRNA expression of antioxidant enzymes, and achieved a cell viability of 91.75% at a concentration of 100 μM [42]. In d-galactose/aluminum chloride (d-gal/AlCl_3_)-induced Alzheimer’s disease (AD)-like rat models, CA extracts significantly improved spatial memory performance and motor dysfunction by alleviating tau pathology and oxidative stress through the activation of the Akt/GSK3β pathway [43]. The combination of ethanolic extracts of *C. asiatica* (CE) and α-tocopherol exhibited synergistic antioxidant effects, with the optimal combined ratio identified as fraction 2/3 across all assays. The complex interactions between CE and α-tocopherol at varying concentrations ultimately led to enhanced antioxidant activity [44].

Other studies have also investigated the neuroprotective effects of CA and its bioactive components against neuroinflammation. In C6 glioma cells, treatment with paracetamol, a commonly used antipyretic drug known for its potential to cause liver toxicity in overdose, resulted in elevated levels of pro-inflammatory cytokines, such as IL-1, TNF-β, and the pro-inflammatory chemokine MCP-1. Interestingly, co-administration of CA extracts (CAM) restored the levels of these inflammatory markers and increased INF-γ mRNA expression, which was later downregulated, indicating a reduction in inflammation [45,46]. Similarly, Hambali et al. demonstrated that the ethanolic extract of *C. asiatica* (SECA) effectively suppressed the production of pro-inflammatory cytokines (TNF-α, IL-6, IL-1β) in LPS-induced microglial cells. Concurrently, SECA enhanced the expression of Nrf2 and HO-1 proteins. The multifunctional regulator nuclear factor erythroid 2-related factor 2 (Nrf2) is known for its cytoprotective properties, as it modulates the expression of genes involved in antioxidant defense, anti-inflammatory responses, and detoxification processes [47,48].

## 4. Therapeutic effects of CA

### 4.1. Therapeutic effects against neurodegenerative diseases

#### 4.1.1. Alzheimer’s disease

Alzheimer’s disease (AD) is a neurodegenerative disorder characterized by brain oxidative stress and progressive cognitive decline [49]. CA fraction was found to successfully repressed cell death and reduced oxygen species production in murine hippocampal cells (HT22) injured by glutamate, an oxidative stress inducer in progression of AD [50]. Fear memory deficits are commonly observed in early-onset Alzheimer’s disease [51]. Oral administration of ECa 233, a standardized extract of CA, surprisingly prevented a cued fear memory deficit and enhanced hippocampal long-term potentiation (LTP) in an Alzheimer’s disease model mouse [52]. Researchers have also evaluated the effectiveness of ethanolic extracts of CA and demonstrated its protective effects against acetylcholinesterase (AChE) activity, an enzyme implicated in AD pathology [53,54].

Disruption of endothelial cell function due to hippocampal Aβ_1–42_ plaque accumulation and tau deposition leads to increased blood-brain barrier (BBB) permeability, which is a contributing factor to neurodegenerative disorders, including Alzheimer’s disease [55]. Asiatic acid (AA) was found to inhibit tau pathology and diminished the expression of cyclin-dependent kinase 5 which involves in the phosphorylation of tau proteins in AD like model rats [43]. In rats with cognitive deficits treated with CA, levels of phosphorylated tau (P-tau) showed a significant decline [56]. These findings enhance our understanding of tau pathology and may inform the development of new therapeutic strategies for Alzheimer’s disease. Dhanasekaran et al. found that prolonged CA treatment alters amyloid β pathology in PSAPP mice, highlighting its antioxidant potential against AD-related neurodegeneration [57]. Additionally, a study reported the anti-apoptotic effects of asiaticoside, showing that it significantly reduced cell growth inhibition and apoptosis by inhibiting the TLR4/NF-κB signaling pathway after 12 h of treatment, suggesting its potential as a clinical agent for AD [58].

Even though the four triterpenes are generally considered as neuroprotective compounds in CA, studies have shown that caffeoylquinic acids (CQAs), another bioactive component found in water extract of CA, also exhibit neurotropic and anti-neurotoxicity abilities. CQAs are noted for their therapeutic potential and are recognized as natural sources of butyrylcholinesterase (BChE), an enzyme that hydrolyzes acetylcholine and is often deficient in Alzheimer’s patients [59]. Additionally, CQAs have demonstrated efficacy in alleviating Aβ aggregation and counteracting cytotoxicity induced by hydrogen peroxide and rotenone [60]. A study in 2020 investigated the neuroprotective effects of CQAs, further confirming their role in the repair and enhancement of age-related cognitive decline associated with CA [61].

#### 4.1.2. Parkinson’s disease

Another age-related neurodegenerative disorder is Parkinson’s disease (PD), which is characterized by the decline of mitochondrial function over time and the subsequent release of harmful reactive oxygen species (ROS), leading to oxidative stress and cellular damage [62]. Studies have shown that ECa 233, a standardized extract of CA, exhibits neuroprotective effects against rotenone-induced mitochondrial dysfunction and neuronal injury [63]. Additionally, Asiaticoside-D demonstrates neuroprotective effects by modulating MAO-A and B levels, thereby mitigating ROS-induced damage in rotenone-treated *Lumbricus terrestris*. This action is considered important in counteracting neurodegenerative changes associated with Parkinsonism [10].

Besides, madecassoside, another compound isolated from CA, has demonstrated neuroprotective effects in models of Parkinson’s disease (PD). It has been shown to alleviate 1-Methyl-4-phenyl-1,2,3,6-tetrahydropyridine (MPTP)-induced Parkinsonism by exerting antioxidant effects, reversing dopamine neuron degeneration, increasing the Bcl-2/Bax expression ratio to regulate apoptosis, and enhancing BDNF protein expression [64,65]. Similar neuroprotective effects have been observed in Sprague-Dawley rats challenged with MPTP, where CA extracts significantly reduced the production of reactive oxygen species (ROS) and alleviated oxidative stress in brain cells, highlighting their potential to combat neurodegenerative disorders like Parkinsonism [66].

Experimental evidence indicates that immune disorders caused by the infiltration of peripheral inflammatory cells can lead to an overactivated immune microenvironment in Parkinson’s disease (PD). Conventional interventions targeting microglia often only provide short-term relief from neuroinflammation, underscoring the need for new strategies to address the chronic neuroinflammation that contributes to Parkinson-like syndromes [67]. Asiaticoside (AS), an anti-inflammatory component derived from CA, has shown promise in MPTP-induced PD mice by suppressing neuroinflammation and inhibiting the activation of the NLRP3 inflammasome. These effects were associated with the rescue of dopaminergic neuron loss and the alleviation of motor dysfunction, suggesting that AS may be a promising inhibitor of PD driven by NLRP3 overactivation [68].

#### 4.1.3. Huntington’s disease

In addition to AD and PD, Huntington’s disease (HD) represents another class of neurodegenerative disorders, distinguished by its unique neuropathological features but sharing some commonalities, such as the nerve cell degeneration of unknown etiology [69]. HD is an autosomal-dominant disorder caused by the expansion of the glutamine tract in the huntingtin (HTT) protein, with pathologies partly associated with mitochondrial dysfunction and oxidative stress-induced neuroinflammation [70–72]. As a potent antioxidant, the hydroalcoholic extract of CA has been shown to reverse 3-Nitropropionic acid (3-NP)-induced lipid peroxidation (LPO), acetylcholinesterase (AChE) activity, and glutathione (GSH) changes in adult zebrafish. This suggests its potential in preventing neurotoxicity and mitigating neurobehavioral deficits associated with HD. Additionally, CA extract has been found to significantly inhibit 3-NP-induced progressive neuronal damage in the brain, improving cognition and locomotor activity [73]. Still, reports on the use of CA’s neuroprotective effects to address HD-related symptoms are limited, further exploration in this area is warranted in future research.

To sum up, CA exerts comprehensive benefits in protection of normal brain function against neuroinflammation and oxidative stress leading to neurodegenerative diseases, such as AD, PD, and HD. However, further research is needed to gain a deeper understanding of CA’s molecular interactive mechanisms and to conduct comprehensive clinical trials to confirm the potential toxicity CA and its derivatives may possess to cause damage to human organs.

### 4.2. Therapeutic effects against chemical substances-induced toxicity

Chemotherapy-induced cognitive dysfunction is considered a poorly understood question in anti-cancer process of patients for which current management strategies are limited [74]. In the 5-fluorouracil induced memory deficits rats, asiatica acid was found to effectively prevent reductions in cell proliferation and cell survival in the subgranular zone (SGZ) of the hippocampal dentate gyrus [75]. This result aligns with Welbat et al., who showed that asiatic acid protects against chemobrain by reducing p21-positive cells and MDA levels in the hippocampus, while upregulating key neurogenesis markers, such as Notch1, (sex determining region Y-box 2) SOX2, (doublecortin) DCX, and (nuclear factor erythroid 2-related factor 2) Nrf2 [76].

Apart from what mentioned above, CA was also observed to display protective abilities against chemical-induced cytotoxicity. Bisphenol A (BPA) is widely used in food packaging and household products, and this chemical compound was found to be related to multiple neurological disorders involving mitochondrial dysfunction and following neuronal apoptosis [77]. Ethanol extract of CA showed positive therapeutic effects on BPA-induced pancreatic islet toxicity mice characterized elevated oxidative stress, heightened levels of proinflammatory cytokines, and reversed mitochondrial dysfunction [78]. Despite the scarcity of research in the field CA’s neuroprotective properties against BPA-induced neurological diseases, the existing evidence suggests that CA’s protective mechanisms against BPA are similar to those observed in other diseases. This opens up promising avenues for future research, encouraging exploration into CA’s potential in mitigating BPA-induced neurological damage and providing new perspectives on its broader neuroprotective properties.

### 4.3. Therapeutic effects against radiation-induced brain injury (RBI)

Radiation-induced brain injury (RBI) represents as a major challenge for cancer patients undergoing cranial radiotherapy, leading to cognitive dysfunction [79]. For instance, Pediatric posterior fossa tumor (PFT) survivors experience long-term cognitive sequelae, including memory disorders, for which irradiation is one of the main risk factors [80]. Research by Baudou et al. demonstrated that radiation can disrupt brain connectivity in memory-related circuits, with specific dose-dependent effects on supratentorial brain structures [81]. Electromagnetic radiation has also been implicated in the development of neurodegenerative diseases by damaging the central nervous system through mechanisms, such as oxidative stress and mitochondrial dysfunction [82]. Previous studies have revealed that long-term radiation exposure led to increased amyloid β (Aβ) deposition in the hippocampus and cognitive impairment [83,84]. These findings suggest that radiation exposure not only poses a risk for RBI but may also contribute to the development of neurodegenerative disorders like AD and PD. These connections underscore the need for further research into protective strategies against RBI and its associated neurological risks.

As mentioned previously, extract of CA efficiently enhanced memory and cognitive function in Alzheimer’s mouse models by activating antioxidant response pathway and releasing the burden of reactive oxygen species (ROS) over-production and protecting normal function of hippocampal mitochondria [85–87]. Besides, the anti-neuroinflammatory property of CA extract for treatment of neurodegenerative diseases is proposed in earlier studies as well, including suppression of pro-inflammatory cytokine/mediator releasing and preventing Aβ_1–42_-induced cytotoxicity and cell apoptosis [58,88,89]. Overall, the comprehensive neuroprotective potentials of CA to boost memory, restore cognition deficits, and improve brain functions are widely recognized, thus making it a possible therapeutic target and an ideal candidate for drug development for radiation-induced brain injury (RBI) patients to help their recovery of cognitive dysfunction and memory impairment.

### 4.4. Therapeutic effects in ameliorating cerebral ischemia-reperfusion injury (CIRI)

Cerebral ischemia-reperfusion injury (CIRI) is characterized by disruption of blood supply to the brain, and then the restoration of blood flow, leading to a cascade of pathological mechanisms and eventually result in neuronal damage and death [90]. The pathogenesis of CI/RI involves multiple aspects, including excessive ROS-induced neuronal oxidative stress [91], inflammation [92], and cell apoptosis [93]. The protective effects of CA and its extracts are discussed in several previous research articles. Asiaticoside (AS), investigated in CIRI both *in vivo* and *in vitro*, showed neuroprotective effects against brain function injury and reduced inflammation level and oxidative stress level *via* the NOD2/MAPK/NF-kappaB signaling pathway [94]. Another effective therapeutic strategy against CIRI is to inhibit the neuroinflammation and modulating microglial overactivation to ameliorate neurological impairment and rescuing cognitive and memory deficits [95]. An early study reported the anti-inflammation effect of CA component through oral administration of AS in memory impaired mice induced by transient cerebral ischemia and reperfusion. It was observed in the study that AS markedly repressed the microglial overactivation and reduced the levels of inflammatory cytokines in hippocampus of the mice, indicating its great potential in alleviating cerebral ischemia reperfusion-induced health problems [96]. Additionally, madecassoside, another component of CA, is also indicated to possess neuroprotective effects against CIRI for it significantly alleviated neuronal apoptosis and oxidative stress, and reduced the levels of pro-inflammatory cytokines in rats [97].

These findings underscore the therapeutic potential of CA and highlight its capacity for treating cerebral ischemia-reperfusion injury (CIRI), which is caused by a complex interplay of cellular and molecular factors. The observed benefits suggest that CA could play a significant role in mitigating the damage associated with CIRI by addressing various pathological mechanisms involved in the injury process.

### 4.5. Therapeutic effects in preventing epilepsy and anti-epileptic drugs-induced cognitive impairment

Epilepsy is a neurological disease characterized by recurrent seizures, which can be acquired following brain insults including trauma, stroke, and infection. It is most commonly found among infants and the elderly and induces multiple harmful consequence containing neuroinflammation, blood-brain barrier (BBB) dysfunction, and oxidative stress in certain region of the brain [98,99]. Despite the widespread use of anti-epileptic drugs (AEDs), approximately one-third of patients develop drug-refractory epilepsy, highlighting the need for alternative therapeutic strategies [100]. AEDs primarily function by suppressing glutamate release and reducing neuronal excitability. Notably, asiatic acid has been reported to presynaptically decrease glutamate release and inhibit spontaneous excitatory postsynaptic currents in the rat hippocampus [101]. This finding indicates asiatic acid to be a potential candidate for development of new therapeutic anti-epileptic drugs. In model mice with kainic acid (KA)-induced seizure, asiatic acid exhibited ability against epilepsy through ameliorating neuronal damage and improving brain cognitive deficits coupled with increasing synaptic and mitochondrial function [11]. Depletion of endogenous antioxidant like glutathione (GSH) and consequent oxidative stress may contribute to status epilepticus (SE), a type of seizure lasting more than 5 min that can have lethal consequences or lead to various neurological disorders, including epilepsy [102–104]. The application of a standardized extract of CA at specific doses produced anticonvulsant effects in lithium/pilocarpine-induced seizures without causing hepatotoxicity or nephrotoxicity, indicating its potential as a new therapeutic option with fewer side effects for the treatment of epilepsy [105].

Cognitive impairment can result not only from recurrent seizures but also as a side effect of long-term use of AEDs. While current AEDs provide protection against epilepsy, they are associated with neurotoxic side effects and cognitive decline [106–108]. The mechanisms by which AEDs influence neurodevelopmental outcomes are not fully understood; however, early studies have demonstrated that AEDs can induce cell apoptosis, alter neurotransmitter environments, and impair synaptogenesis, which are thought to contribute to cognitive deficits in patients [109]. An early study suggested that the aqueous extract of CA could serve as an adjunct to AEDs, offering additional protection against AED-induced cognitive impairment [110]. Evaluation of interaction profile of hydroalcoholic extract of *C. asiatica* (HECA) with antiepileptic drugs indicated that co-administration of HECA could enhance the therapeutic efficacy of valproate and phenytoin by attenuating seizure induced oxidative stress and cognitive impairment [111].

Overall, utilizing CA and its bioactive triterpene compounds as therapeutic agents present a novel and promising future in the treatment of epilepsy.

### 4.6. Therapeutic effects in relieving anxiety- and depression-like symptoms

In addition to its effectiveness in protecting against neurodegenerative disorders, such as AD and PD, CA has also been shown to mitigate anxiety- and depression-related behaviors.

The earliest study could be traced back to 2006 by Wijeweera et al., which assessed the effects of different CA plant materials and revealed that methanol and ethyl acetate extracts of CA, as well as the pure asiaticoside, displayed anxiolytic activity. Notably, asiaticoside did not alter locomotor activity, indicating that these compounds do not have sedative effects in rodents [112]. Sleep deprivation (SD), characterized by inadequate or poor sleep, can lead to anxiety-like behavior. Previous research has highlighted CA’s protective effect against SD-induced anxiety-like behavior, potentially involving a nitric oxide (NO) modulatory mechanism [113]. Ling et al. further demonstrated that madecassoside (MA), a triterpenoid saponin component of CA, improved sleep-wake cycle rhythms and synaptic plasticity in protein L-isoaspartyl methyltransferase (PIMT/PCMT1) deficient mice. These findings suggest MA’s potential in alleviating anxiety-associated symptoms and cognitive deficits [114]. An earlier report also noted the anxiolytic effects of ECa 233, a standardized extract of CA, observing positive impacts on both acutely and chronically stressed animals. The writers speculated the anxiolytic effect of CA could mainly be attributed to madecassoside and asiaticoside [115]. This conclusion reaches agreement with previous reports on effects of CA in attenuating anxiety-like behaviors, prospecting the likely essential areas to which future research should attach more attention.

Depression is a common mental disorder that can negatively impact psychosocial function and quality of life. CA demonstrates promising anti-stress activity in reserpine-induced zebrafish stress-like zebrafish models, which plays a vital role in inhibiting the pathogenesis of depression [116]. While conventional antidepressant medications often come with unwanted side effects, traditional herbal medicines, such as CA are generally well tolerated. Holvoet et al. demonstrated that chlorogenic acids in CA extracts exert their effects through calcineurin, a phosphatase involved in regulating synaptic transmission and plasticity. This interaction contributes to resilience against stress-induced phenotypes and ameliorates depression-associated symptoms [117]. Additionally, asiaticoside from CA has been identified as a significant antidepressant and anti-inflammatory agent in a mouse model of chronic unpredictable mild stress (CMS), with its effects mediated through the regulation of the cAMP/PKA signaling pathway [118].

In summary, studies on CA and its active components have demonstrated their therapeutic potential in treating neurodegenerative diseases (NDs), chemical- and radiation-induced brain damage, cerebral ischemia-reperfusion injury (CIRI), reducing brain injury caused by epilepsy and prolonged use of anti-epileptic drugs, as well as counteracting depression and anxiety-like behaviors across various models (Table 1). Though experimental evidence suggests significant potential for CA in addressing multiple neurological disorders and enhancing patients’ quality of life, further research and additional clinical trials are necessary to fully assess its potential biotoxicity against human organs.

**Table 1.** Studies of therapeutic effects of CA and its active compounds.

Type of disease		Animal models, drug doses, and timing	Effects of application	References
Neurodegenerative disease	Alzheimer’s disease	Mice with aluminum chloride (AlCl_3_)-induced amyloid pathology were treated orally with AA (75 mg/kg b.w.) for 7 weeks	Phosphorylation of CDK 5-enzyme induced-tau pathology↓	[43]
		Triple transgenic Alzheimer’s disease (3xTg-AD) model mice were treated with ECa 233 (10, 30, and 100 mg/kg) for 30 consecutive days	Brain-derived neurotrophic factor (BDNF)↑, tyrosine receptor kinase B (TrkB) and its network proteins↑, extracellular signal-regulated kinase 1 and 2 (ERK1 and 2)↑	[52]
		Aβ_1–42_-induced AD mice were administrated with AS (40 mg/kg) intragastrically within 2–14 days	Amyloid-β pathology↓, reduced cognitive impairment by inhibiting p38 MAPK pathway and promoting synaptic repair	[119]
	Parkinson’s disease	Rotenone-induced parkinsonism rats were given either vehicle or ECa233 (10, 30, and 100 mg/kg) orally for 20 consecutive days	Distances, number and intensity of dopaminergic neurons↑, mitochondrial complex I activity↑, MDA↓, SOD↑, catalase expression↑	[63]
		MPTP-induced parkinsonism model mice were given madecassoside for 7 days before injection of MPTP on the 7th day, and then treated continuously with madecassoside for the following 14 days	Depletion of dopamine↓, MDA↓, glutathione (GSH)↑, Bcl-2/Bax ratio↑, protein expression of BDNF↑	[65]
		Mice with LPS-induced neuroinflammation were administrated with plant extracts including CA (200 mg kg^−1^) orally from day 1 to day 12	Activation of glial cells↓, TNF-α↓, IL-1β↓, C3 complement proteins overproduction↓	[120]
	Huntington’s disease	Zebrafish with 3-NP induced Huntington’s like symptoms received CA treatment (80 and 100 mg/L) daily for up to 28 days in water	Lipid peroxidation (LPO)↓, AChEs↓, nitrite↓, tumor necrosis factor-α and interleukin-1β↓, glutathione (GSH)↑	[73]
Chemical substances-induced toxicity	Chemotherapy-induced cognitive dysfunction	Mice treated by 5-FU chemotherapy received oral administration of AA (30 mg/kg) for 20 days before, during and after 5-FU treatment separately	Notch 1↑, sex determining region Y-box 2 (SOX2)↑, nestin↑, doublecortin (DCX)↑, nuclear factor erythroid 2-related factor 2 (Nrf2)↑, p21 positive cells↑, MDA↑	[76]
	Bisphenol A (BPA)-induced toxicity	Mice with BPA-induced toxicity were treated with CA (200 and 400 mg/kg body weight) for 21 days	Oxidative stress↓, proinflammatory cytokines↓, loss of mitochondrial membrane potential (MMP)↓, apoptosis↓, abnormal cell cycle↓	[78]
Radiation-induced brain injury (RBI)		Mice with LPS induced memory and learning deficits were administrated with CA ethanol extract (200 mg/kg) orally for 14 days	Spatial memory↑, learning deficits↓, proinflammatory responses↓	[89]
Cerebral ischemia-reperfusion injury (CIRI)		CIRI model mice induced by middle cerebral artery occlusion were treated orally with different concentrations of asiaticoside at doses of 20, 40, and 60 mg/kg	Nervous function injury↓, brain edema↓, cell apoptosis↓, protein expressions of the NOD2/MAPK/NF-kappaB signaling pathway↓	[94]
Epilepsy	Epilepsy-induced cognitive impairment	Mice with kainic acid (KA)-induced seizure were treated with asiatic acid (10 and 50 mg/kg) intraperitoneally 30 min prior to KA injection	Neuronal damage↓, calpain activation↓, protein kinase B (AKT) activation↑, synaptic proteins and vesicles↑, mitochondrial damage↓	[11]
	Anti-epileptic drugs-induced cognitive impairment	Mice with valproic acid (VPA)-induced neurogenesis impairments were administrated orally with asiatic acid (30 mg/kg/day for 28 days)	Spatial working memory↑, cell proliferation↑, cell survival in the subgranular zone (SGZ) of the hippocampal dentate gyrus (DG)↑, Ki-67 and BrdU positive cells↑	[121]
		Mice with Pentylenetetrazole (PTZ)- and maximal electroshock seizure (MES)-induced seizures received co-administration of valproate (VPA) and phenytoin (PHT) with HECA respectively	Oxidative stress↓, cognitive impairment↓, serum levels of VPA and PHT↑	[111]
Anxiety and depression	Anxiety	Mice were subjected to acute stress or chronic immobilization stress respectively and treated with ECa 233, and later anxiolytic effect was assessed by an elevated plus maze (EPM), a dark-light box and an open-field tests	Stress↓, body weight↑, serum corticosterone level↓	[115]
		Mice with sleep deprivation (SD) induced-anxiety like behavior were treated with CA (150 and 300 mg/kg) alone and in combination with NO modulators for 8 days, starting five days before 72-h SD exposure	Locomotor activity↑, oxidative stress↓, anxiety↓, TNF α level↓	[113]
		Mice with protein L-isoaspartyl methyltransferase (PIMT/PCMT1) deficiency-induced anxiety related to neurodegenerative diseases were treated with madecassoside i.p. once two days (2 mg/kg) for a week	Circadian rhythms sleep-wake cycle↑, sleep disturbance↓, synaptic plasticity↑	[114]
	Depression	Zebrafish with reserpine-induced stress-like behaviors were treated with CA and its triterpenoids compounds	Freezing duration↓ and total traveling distance ↑in the open field test, locomotion↑, metabolic pathways involved in anti-stress effect↓	[116]
		Mice with maternal deprivation (MD)-induced depression were administrated with CA and its active compounds (10 mg/kg) for 14 days	Depressive-like behaviors↓, inflammation in the hippocampus↓, oxidative stress in the serum↓	[122]

## 5. Computational studies on neuroprotective molecular mechanisms of CA

CA and its extracts demonstrate broad neuroprotective effects through anti-inflammatory, anti-apoptotic, and antioxidant pathways. A deeper understanding of the molecular targets of its bioactive constituents is essential for elucidating its therapeutic potential in various neurological disorders and may serve as a valuable reference for the development of targeted pharmaceuticals. Computational approaches like network pharmacology and molecular docking can be used to effectively map CA’s target-compound interactions, revealing therapeutic mechanisms and potential neuroprotective applications.

Through network pharmacology, Zhao et al. identified twenty-five potential active ingredients in CA for anti-angiogenesis with 19 core targets, including MAPK1, AKT, etc. Their findings suggest CA’s significant potential as a candidate for cancer therapy [123]. Ansari et al. further demonstrated CA’s ability to modulate neuroinflammatory and neurodegenerative processes through its effects on AKT, MAPK, TNF-α, and NF-κB signaling networks [124]. Another metabolomics and network pharmacology study identified shared neuroprotective metabolites between *B. monnieri* and CA, primarily targeting MAPK, mTOR, and PI3K-AKT pathways, consistent with previous findings [40].

In the context of neurodegenerative diseases, asiaticoside, one of the bioactive constituents of CA, has been shown to exert neuroprotective effects in an MPTP-induced Parkinson’s disease (PD) rat model. Through computational modeling, NLRP3 was identified as a key molecular target, and the NOD-like receptor signaling pathway was recognized as a critical mechanism underlying its therapeutic action [68]. The pathological mechanisms of Alzheimer’s disease involve the dysfunction of acetylcholinesterase (AChE) and β-secretase (BACE1). Utilizing computer-aided discovery approaches, Shah et al. identified CA as a rich source of dual inhibitors targeting both AChE and BACE1. This finding further highlights the remarkable potential of CA in the treatment of neurodegenerative diseases [125].

**Figure 3.**
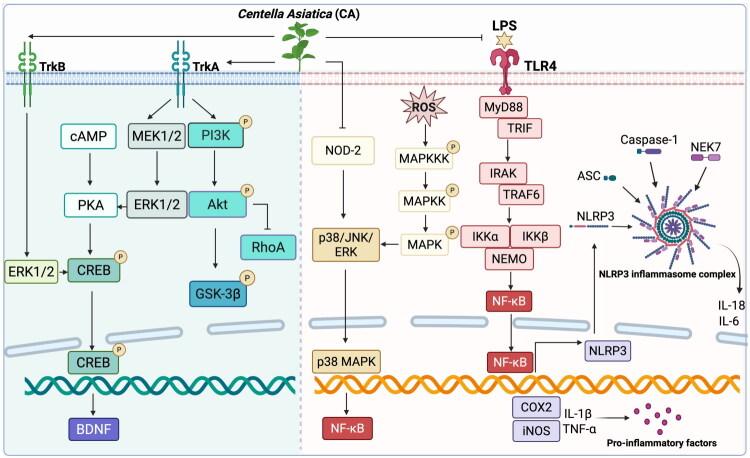
The cellular mechanisms and related signaling pathways involved in the neuroprotective effects of CA. Created with BioRender.com.

Based on the discussion of the therapeutic effects of CA in Section 4 and the molecular mechanism analysis from computational studies in Section 5, the key signaling pathways involved in the neuroprotective actions of CA have been summarized and illustrated in Figure 3 for reference.

## 6. CA and the blood-brain barrier: permeation potential and drug delivery systems

In the preceding discussion, we have highlighted the positive therapeutic effects of CA in the treatment of neurodegenerative diseases. Among the existing research findings, how to efficiently cross the blood-brain barrier (BBB) remains a significant challenge for drug delivery and current therapeutic strategies in the treatment of neurodegenerative diseases [126].

Key bioactive compounds in CA demonstrate distinct blood-brain barrier (BBB) penetration capabilities. Asiatic acid exhibits optimal physicochemical properties for passive diffusion (logP = 5.7, MW < 500 Da, ≤5 H-bond donors/acceptors), consistent with Lipinski’s Rule of Five [127]. *In vitro* studies using porcine BBB models confirm its efficient transport alongside asiaticoside and madecassoside, all without evidence of toxicity. While asiatic acid’s lipophilicity facilitates membrane diffusion, the glycosylated structures of asiaticoside and madecassoside suggest alternative transport *via* transcytosis mechanisms [128]. These differential penetration pathways collectively support CA’s neuroprotective potential in neurodegenerative disorders. Future studies involving bidirectional permeability assays could further elucidate the transport mechanisms of these compounds [15].

Building upon this, various drug delivery strategies have been explored to further enhance the brain-targeting efficiency of asiatic acid. Designed materials, such as solid lipid nanoparticles (SLNs) possess a promising potential as a drug delivery system to carry lipophilic drugs across BBB. Studies proved that asiatic acid-loaded solid lipid nanoparticles exerted anti-cancer efficacy against glioblastoma, confirming the potential of exploiting these nanoparticles for brain cancer therapy [129]. As previously mentioned, improving the solubility and bioavailability of poorly water-soluble drugs remains a key challenge, and one promising strategy involves pharmacosomes—amphiphilic drug-phospholipid complexes that self-assemble into lipid bilayers in aqueous environments, enhancing both solubility and oral bioavailability. For instance, a phospholipid-based complex of standardized Centella extract (SCE) achieved a 12-fold increase in aqueous solubility, significantly improving systemic absorption and demonstrating beneficial effects on spatial learning and memory in aged mice [36].

Besides, the intranasal route of drug administration offers an opportunity to bypass the BBB and deliver the therapeutic compounds directly into the brain. Haasbroek-Pheiffer et al. showed that CA components exhibited higher permeation across the olfactory epithelial tissue than across the respiratory epithelial tissue, and therefore demonstrating the possibility for direct nose-to-brain delivery of these psychoactive phytochemicals [130]. A newly developed polymer hybrid nasal nanocomposite for enhanced delivery of CA to the CNS was reported by Haroon et al. of which thiolated chitosan was complexed with CA to form a composite using EDAC hydrochloride. The nanocomposite showed no signs of nasal ciliotoxicity and good permeation and lesser toxicity compared to the free drug [131]. Another study also demonstrated the nose-to-brain delivery of asiatic acid in SLN could be an essential strategy to treat the early stage of AD with improved bioavailability and enhanced penetration of CA compounds in the brain [132].

## 7. Future research directions

The neuroprotective properties of CA have been extensively reported in the previous literature. To further enhance its clinical application potential and fully exploit its bioactivities, future studies should expand our understanding of the pharmacokinetic properties of active compounds in CA or its extracts through various administration routes. These insights are essential for clarifying the *in vivo* efficacy of the compounds.

In addition, several active compounds in CA are macromolecules, which typically exhibit low water solubility and poor intestinal absorption, thereby limiting their oral bioavailability. Future research should focus on improving their solubility and bioavailability through advanced drug delivery systems (e.g. nanoparticles, intranasal formulations) which is critical for clinical application. Finally, future studies should focus on elucidating the precise molecular mechanisms of CA’s neuroprotection, particularly its cell-type-specific effects and signaling pathways, which will deepen our understanding of the cellular mechanisms by which CA exerts its effects in various neurological diseases and may inspire the development of targeted therapeutics.

## 8. Conclusions

*Centella asiatica* (L.) Urb. is an herbaceous plant with potential therapeutic effects and significant value for drug development. The medicinal properties of CA and its bioactive components are primarily attributed to their antioxidant, anti-inflammatory, and anti-apoptotic characteristics, as demonstrated in numerous studies. Furthermore, CA and its various extracts exhibit promising therapeutic effects in treating various neurodegenerative diseases, such as Alzheimer’s, Parkinson’s, and Huntington’s diseases. Additionally, they hold potential in combating chemical toxicity, radiation, cerebral ischemia-reperfusion injury (CIRI), epilepsy, and cognitive impairments induced by antiepileptic drugs, as well as in alleviating symptoms related to anxiety and depression. In the final part of this review, we examine the favorable properties exhibited by certain components of CA in crossing the blood-brain barrier and discuss various drug delivery mechanisms developed in related studies. Future research should focus on elucidating the specific cellular and molecular mechanisms underlying the neuroprotective effects of CA and its derivatives, investigating their potential synergistic effects and possible biotoxicity, and exploring strategies to enhance their bioavailability and absorption in the human body, thereby providing new avenues for drug development.

## Data Availability

Data sharing is not applicable to this article as no data were created or analyzed in this study.
